# Flower‐Like Colloidal Particles through Precipitation Polymerization of Redox‐Responsive Liquid Crystals

**DOI:** 10.1002/anie.202111521

**Published:** 2021-11-17

**Authors:** Xiaohong Liu, Mohammad‐Amin Moradi, Tom Bus, Michael G. Debije, Stefan A. F. Bon, Johan P. A. Heuts, Albert P. H. J. Schenning

**Affiliations:** ^1^ Stimuli-Responsive Functional Materials and Devices Department of Chemical Engineering and Chemistry Eindhoven University of Technology Groene Loper 3 5612 AE Eindhoven The Netherlands; ^2^ Institute for Complex Molecular Systems Eindhoven University of Technology Groene Loper 3 5612 AE Eindhoven The Netherlands; ^3^ Laboratory of Physical Chemistry Department of Chemical Engineering and Chemistry Eindhoven University of Technology Groene Loper 3 5612 AE Eindhoven The Netherlands; ^4^ Department of Chemistry The University of Warwick Coventry CV4 7AL UK; ^5^ Supramolecular Polymer Chemistry group Department of Chemical Engineering and Chemistry Eindhoven University of Technology Groene Loper 3 5612 AE Eindhoven The Netherlands

**Keywords:** flower-like particles, liquid crystal, precipitation polymerization

## Abstract

We report on the synthesis of monodisperse, flower‐like, liquid crystalline (LC) polymer particles by precipitation polymerization of a LC mixture consisting of benzoic acid‐functionalized acrylates and disulfide‐functionalized diacrylates. Introduction of a minor amount of redox‐responsive disulfide‐functionalized diacrylates (≤10 wt %) induced the formation of flower‐like shapes. The shape of the particles can be tuned from flower‐ to disk‐like to spherical by elevating the polymerization temperature. The solvent environment also has a pronounced effect on the particle size. Time‐resolved TEM reveals that the final particle morphology was formed in the early stages of the polymerization and that subsequent polymerization resulted in continued particle growth without affecting the morphology. Finally, the degradation of the particles under reducing conditions was much faster for flower‐like particles than for spherical particles, likely a result of their higher surface‐to‐volume ratio.

## Graphical abstract

Flower‐like particles of a redox‐responsive liquid crystalline mixture prepared by precipitation polymerization are reported. The influences of monomer composition, polymerization temperature, and solvent on the particle shape are investigated. The shapes formed in the early stage of polymerization remain unchanged. Under reducing conditions, the flower‐like particles are degraded much more rapidly than the spherical particles due to their higher surface‐to‐volume ratio.

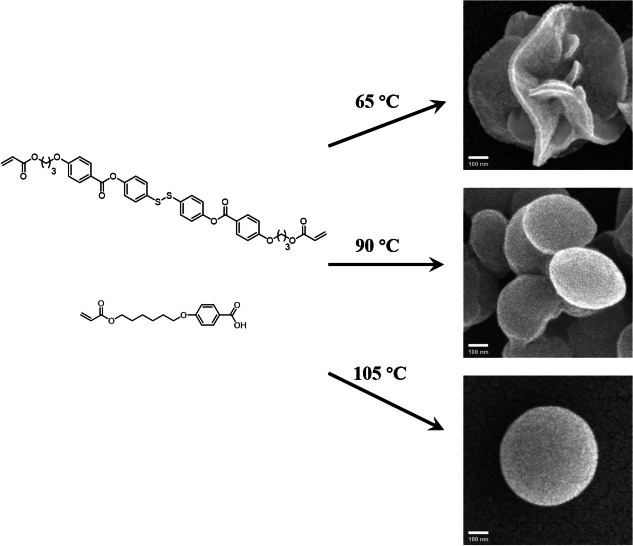

## Introduction

Shape‐anisotropic colloids are of great interest across a wide range of scientific disciplines and application areas. When we look at particles dispersed in a liquid, their geometry may impact individual particle motions[^1^, ^2^, ^3^, ^4^] and interaction with other objects,^[5]^ with shape entropy influencing assembly behavior in crowded environments.[^6^, ^7^] Surface roughness alters the hydrodynamics and rheological behavior of particle dispersions.^[8]^ Shape‐anisotropy affects particle interaction with light,^[9]^ leading to advances in plasmonic nanosensors,^[10]^ opacifiers,^[11]^ and switchable, full‐color reflective dispersions.^[12]^ The shape and surface roughness^[13]^ of colloidal particles alters their wetting characteristics, with applications in fluid repellent coatings,[^14^, ^15^, ^16^] and Pickering emulsions.[^17^, ^18^] Particle geometry is also emerging as an important factor in biological cell interactions and uptake.[^19^, ^20^]

Despite the plethora of available anisotropic colloids, it remains challenging to fabricate non‐spherical polymer particle colloids. One approach is to physically deform spherical thermoplastic polymer particles by stretching above their glass transition temperature,[^21^, ^22^, ^23^] or via template‐guided 2D[^24^, ^25^] and 3D^[26]^ partial film formation. Templated or confined geometric synthetic routes, such as photolithography,[^27^, ^28^] particle replication in non‐wetting templates (PRINT),^[29]^ and droplet‐based microfluidic routes,[^30^, ^31^] can also bring results. Although these methods offer direct control of morphology, they face difficulties as the dimension of the particles decreases to sub‐micron range; in addition, the low productivity and stringent preparation conditions remain challenging.

Bottom‐up, template‐free synthetic routes, though impressive, still commonly result in rounded morphologies, such as dumbbells,[^32^, ^33^] patchy^[34]^ and raspberry‐type armored particles,^[35]^ or more exotic “octopus ocellatus” particles.^[36]^ Even polymerization‐induced self‐assembly (PISA) techniques usually lead to rounded spherical, cylindrical, or vesicular shapes.^[37]^ To obtain suprastructures with angular shapes, crystallization is often employed. Indeed living‐crystallization driven self‐assembly^[38]^ allows for this angular complexity in particle geometry. Alternatively, by using a liquid crystalline monomer, colloidal suprastructures obtained through PISA by radical polymerization showed a more angular appearance, characteristic of crystallinity.^[39]^

In the 1960s, highly crystalline polyimide powders of intricate morphology were prepared through imidization of polyamide‐acids.[^40^, ^41^] An illustrative example of this was 1–5 μm average diameter polyimide particles with sheaf or coral‐like morphologies.^[42]^ Synthesis of conjugated polymers under heterophase conditions produce crystalline cylindrical‐shaped particles, also with sharp edges.^[43]^ This concept of preparing polymers which phase separate from and precipitate out of solution into particles which then crystallize is intriguing, especially when providing a route to angular shapes, especially if control of the particle size distribution can be achieved. Whereas precipitation polymerization often shows a lack of control over particle formation and growth, when the conditions are right it is possible to prepare monodisperse particles, for example in the free radical precipitation polymerization of divinylbenzene in acetonitrile.^[44]^

Previously, we reported on the preparation of spherical polymer particles via precipitation polymerization of a smectic a liquid crystalline (LC) monomer mixture consisting of a cross‐linker and benzoic acid hydrogen‐bonded dimers.^[45]^ In this paper, we report the preparation of monodisperse, “flower‐like” particles via precipitation polymerization of a LC monomer mixture. The monomer mixture consists of the same benzoic acid‐functionalized acrylates as used before but the cross‐linker is replaced by a redox‐responsive disulfide‐functionalized diacrylate. It is found that the introduction of a minor fraction of the disulfide‐functionalized diacrylates (≤10 wt %) induces the formation of a flower‐like morphology. The shape of these particles can be tuned from flower‐like to spherical simply by elevating the polymerization temperature, while the solvent itself mainly influences the particle size. The flower‐like morphology already forms during the early stages of the polymerization, after which particle growth occurs. Degradation of the particles by breaking the disulfide groups under reducing conditions is dramatically accelerated in the flower‐like particles, attributed to their high surface‐to‐volume ratio.

## Results and Discussion

**Figure 1:**
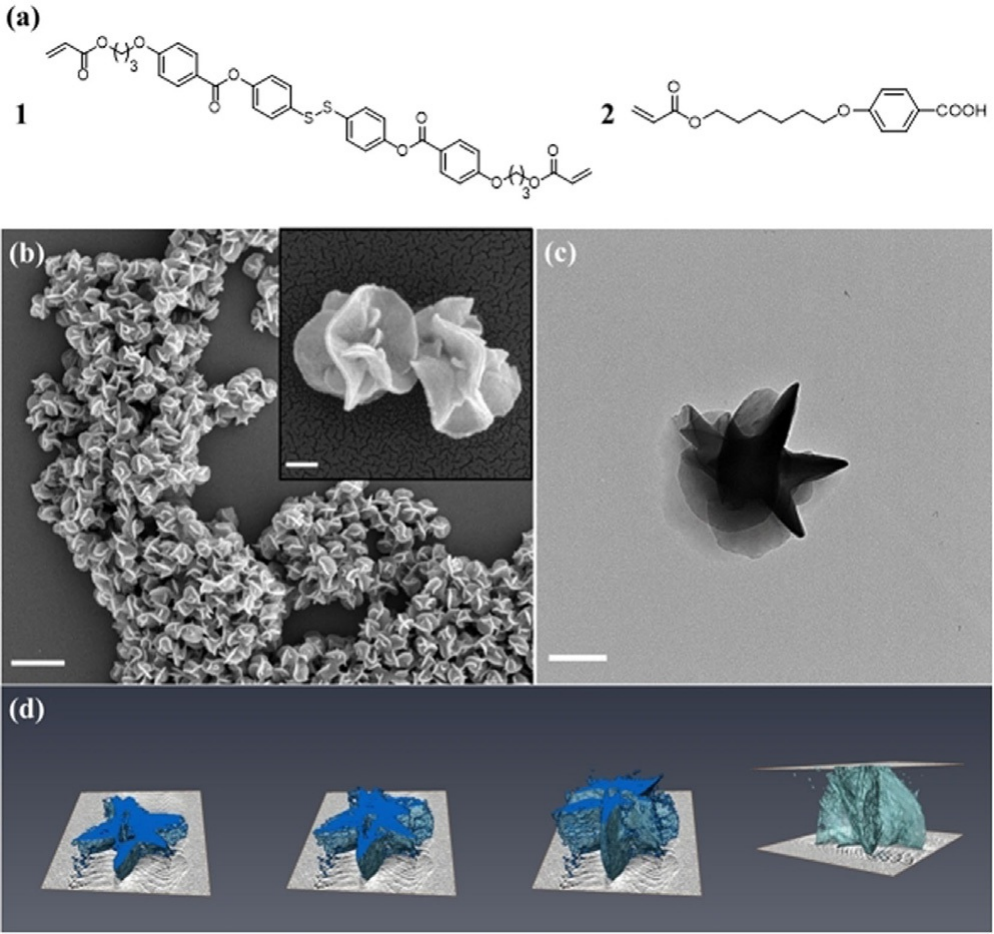
a) LC monomer mixture used to prepare LC polymer particles (weight ratio **1**:**2**=10:90). b) SEM images (scale bar=2 μm (larger image) and 200 nm (inset)) and c) TEM image (scale bar=500 nm) of the flower‐like particles prepared in phenyl acetate at 65 °C. d) 3D tomography of the flower‐like particles.

Monomer **1** (Figure 1 a) was synthesized by esterification of the disulfide core and benzoic acid‐functionalized acrylates (see the supporting information (SI) for details).^[46]^ The LC monomer mixture was then prepared by mixing monomers **1** and **2** (Figure 1 a) in a 10/90 weight ratio. Differential scanning calorimetry (DSC) shows an exothermal peak at around 92 °C, corresponding to the LC‐to‐isotropic transition, with a peak at approximately 50 °C, corresponding to the LC‐to‐crystalline transition (Figure S5). The monomers and thermal initiator were dissolved in a solvent, polymerized overnight, and washed with ethanol to yield the final particles (for experimental details, see the SI). Initial studies were conducted in phenyl acetate at 65 °C based on our earlier results using a similar LC monomer mixture containing a different cross‐linker, vide supra.^[45]^ The resulting polymer particles were flower‐like, with “petal” moieties on the surfaces (Figure 1 b and c). The average diameter of the particles was ≈750 nm, with a coefficient of variation (CV) of 7.2 % and an average circularity of 0.59. 3D Tomography was used to investigate the particle morphology in more detail (Figure 1 d) confirming the flaky moieties throughout the particles. The glass transition temperature (*T*_g_) of the LC polymer particles was determined by DSC and found to be around 82 °C (Figure S6). The flower‐like polymer particles do not change their shape when heated above *T*_g_ at 105 °C overnight (Figure S7), confirming the permanent shape of the particles.

**Figure 2:**
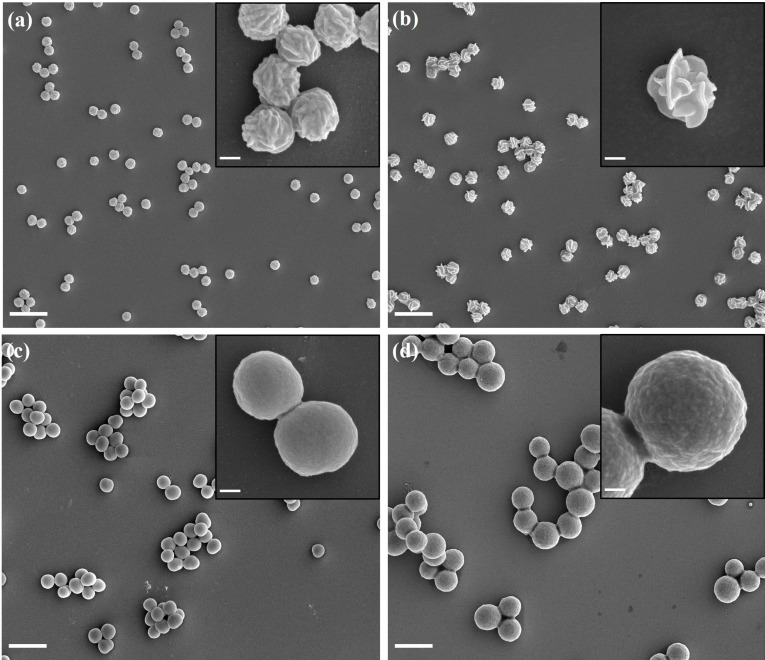
SEM images of particles prepared with monomer **1** and **2** in weight ratios a) 0/100, b) 5/95, c) 20/80, and d) 50/50 in phenyl acetate at 65 °C. Scale bar=2 μm in all large images and 200 nm in all insets.

The concentration of cross‐linking monomer **1** was varied to investigate its impact on the final shape of the particles. As shown in Figure 2 a, without monomer **1**, fairly spherical particles with rough surfaces were obtained. Flower‐like particles were obtained when 5 % or 10 % of monomer **1** was used (Figure 1 and Figure 2 b). Further increasing the weight ratio of monomer **1** to above 20 % resulted in particles with nearly spherical shapes, and no particles were formed at all when pure monomer **1** was used. Meanwhile, the diameter of the particles increased from 500 nm to 1.3 μm with increasing monomer **1**. It is concluded that the introduction of a minor amount of monomer **1** (≤10 wt %) can induce the flower‐like shape, while further increasing the amount of monomer **1** led to spherical particles, probably because the rapid cross‐linking prevented the rearrangement of the polymer chains or monomer **1** disrupted the molecular order. The monomer composition of 10/90 resulted in the most pronounced flower‐like morphologies and was used for the investigations in the remainder of this research.

Since previous publications indicated that the polymerization conditions, including the solvent and polymerization temperature, may have profound influence on particle morphologies, LC polymer particles were prepared using a series of solvent mixtures and polymerization temperatures to reveal the impact of these conditions.[^45^, ^47^]

**Figure 3:**
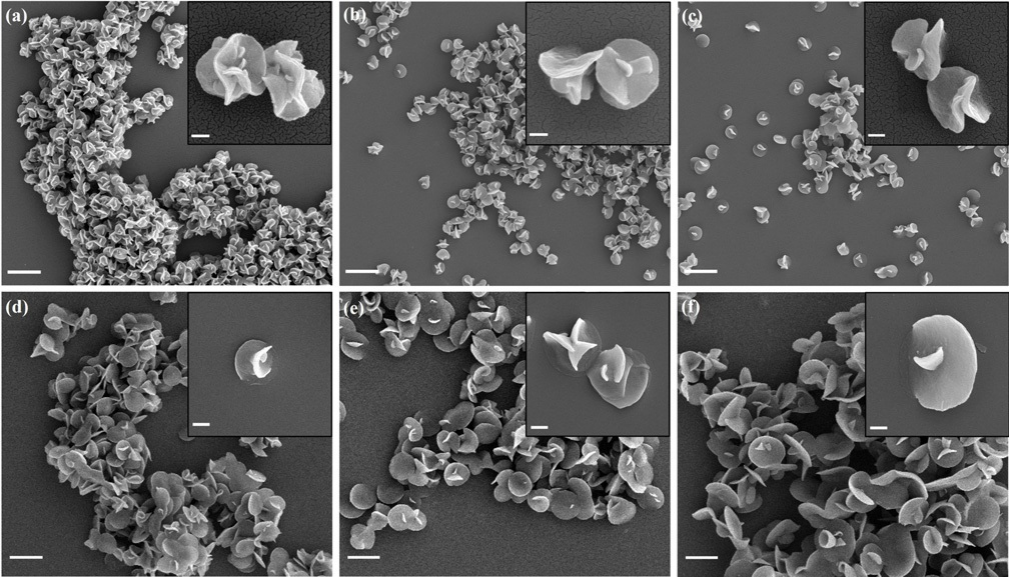
Particles prepared at 65 °C in mixtures of phenyl acetate and ethyl acetate. The volume ratios of phenyl acetate and ethyl acetate are a) 100/0, b) 80/20, c) 60/40, d) 40/60, e) 20/80, and f) 0/100, respectively. Scale bar=2 μm in the large images; scale bar=200 nm (inset of (a), (b), and (c)) and 1 μm (inset of (d), (e), and (f)).

Precipitation polymerization was carried out in solvent mixtures of ethyl acetate and phenyl acetate at different volume ratios at 65 °C to investigate the impact of the solvent on the particles, since in a previous publication, monodisperse LC polymer particles were prepared in these two solvents.^[45]^ As shown in Figure 3, particles prepared from all solvent mixtures are flower‐like. Particles prepared in phenyl acetate‐rich solvent mixtures all have similar average diameters of 750 nm, while particles prepared in ethyl acetate‐rich solvent mixtures show increasing diameters from 1.2 μm to 1.8 μm, with apparently smoother, less flaky surfaces. The increase in diameter is likely due to the higher solubility of the polymer in ethyl acetate, which results in slower and less nucleation and thus larger particles.^[45]^ These results suggest that the particle size and morphology are both affected by the solvent‐polymer interaction.

**Figure 4:**
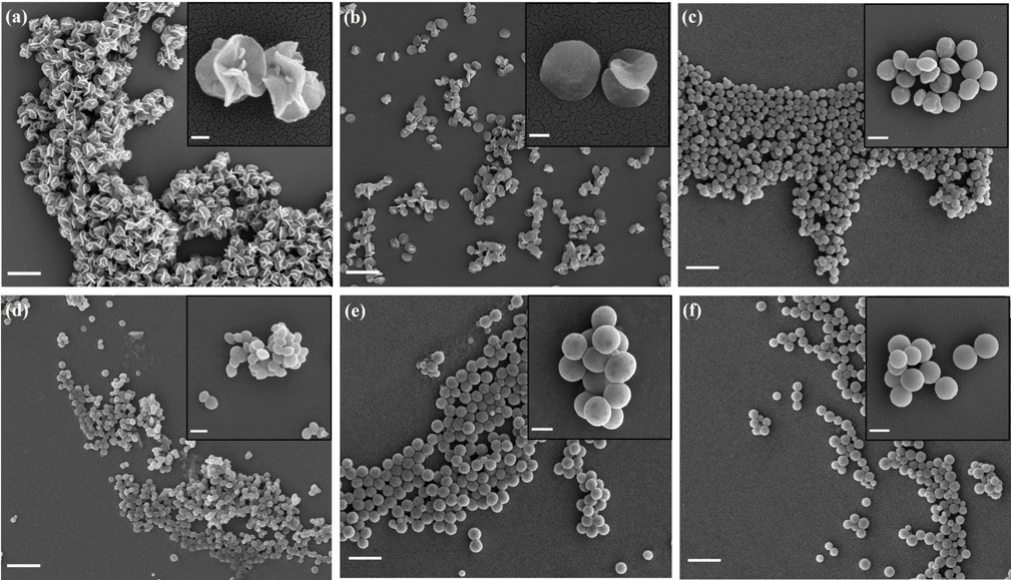
Particles prepared in phenyl acetate at 65 °C (a), 75 °C (b), 85 °C (c), 90 °C (d), 95 °C (e), and 105 °C (f). Scale bar=2 μm (larger image) and 200 nm (inset) in (a) and (b); Scale bar=5 μm (larger image) and 500 nm (inset) in (c), (d), (e), and (f).

Another critical parameter in precipitation polymerization is the polymerization temperature, which not only affects the solvent‐polymer interaction, but also the liquid crystal phase and chain mobility in the polymerizing particles. Polymerization was performed in pure phenyl acetate at a series of temperatures from 65 °C to 105 °C. As shown in Figure 4, the average diameters lie between 400 nm (90 °C) and 750 nm (65 °C). The morphology of the particles prepared at 65 °C and 75 °C were flower‐like, while the particles prepared at 85 °C and 90 °C were disk‐shaped, and particles prepared at 95 °C and 105 °C were spherical. This result indicates that the polymerization temperature has a profound influence on the final morphology and the flower‐like morphology is stable due to cross‐linking.

**Figure 5:**
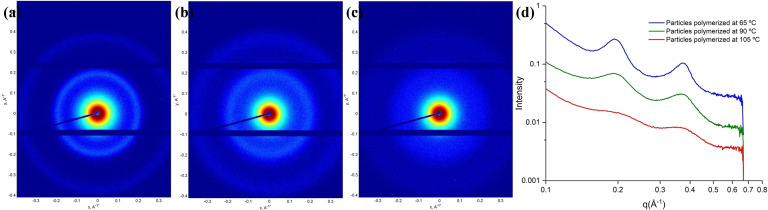
MAXS patterns of particles prepared at 65 °C (a), 90 °C (b), and 105 °C (c). d) MAXS 1D profile derived from (a) to (c). The first order and second order peaks corresponding to a layer spacing of 3.2 nm.

To investigate the LC order in the particles, flower‐like (65 °C), disk‐shape (90 °C), and spherical (105 °C) particles were treated with 10 mM KOH to introduce potassium ions and increase contrast prior to medium‐angle X‐ray scattering (MAXS) measurements.^[45]^ The first and second order peaks correspond to a layer spacing of 3.2 nm which can be attributed to the lamellar order of the molecules as found earlier.^[48]^ The intensity of the peaks was found to decrease with increasing polymerization temperature, indicating that the order was decreased at elevated temperatures (Figure 5). Although the thermotropic LC mixture is in the isotropic state the polymer chains might align resulting in the layered structure in the differently shaped particles.

**Figure 6:**
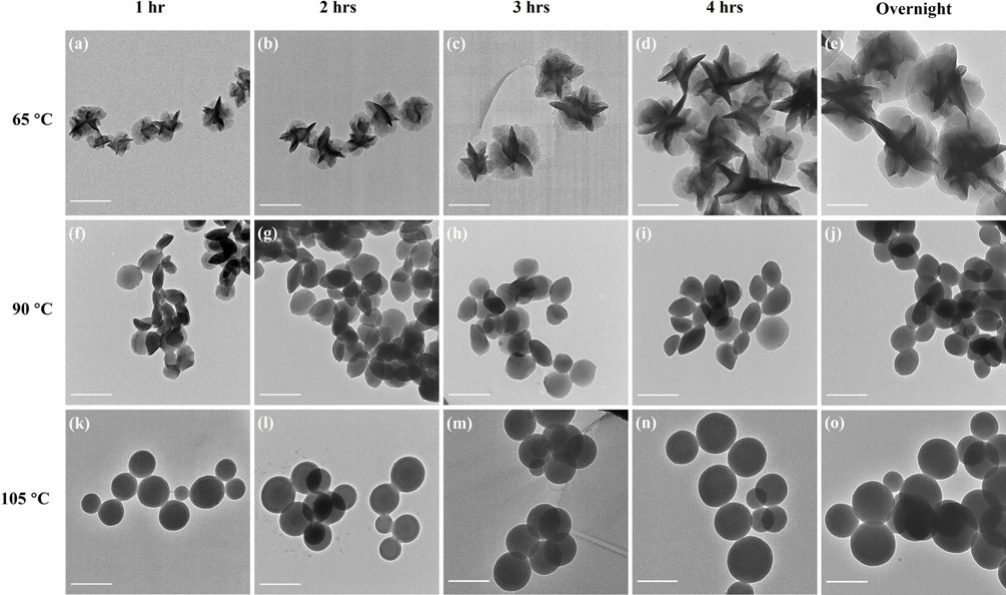
Time‐resolved TEM images of the particles prepared in phenyl acetate at different polymerization temperatures and times (scale bar=500 nm).

The evolutions of the morphologies of the particles were investigated by time‐resolved TEM. A small volume of the polymerization mixture was withdrawn from the flask every hour and washed with ethanol to remove the unreacted monomers for time‐resolved TEM. As shown in Figure 6 a to e, the particles prepared at 65 °C showed flower‐like morphologies from the outset (1‐hour polymerization) which continued to grow in size as the polymerization progressed. The initial diameter was approximately 300 nm, increasing to 750 nm overnight. Both spherical and ellipsoidal shapes were initially observed in the 90 °C polymerization, eventually leading to the formation of disk‐shaped particles (Figure 6 f to j). For polymerizations carried out at 105 °C, only spherical particles were observed throughout the entire polymerization process (Figure 6 k to o). In all cases, the increase in particle size is accompanied by an increase in monomer conversion (see SI for details). In summary, the shape of the LC particles is already formed at the initial stage of the polymerization, and subsequent polymerization only results in the growth of the particles with no change in morphology.

Based on these results, we postulate the formation of the flower‐like morphology was the combined result of liquid crystalline molecular order and limited chain mobility. The liquid crystalline molecular order is reduced and the chain mobility is increased at elevated temperatures, resulting in the rounded morphology of the particles.

**Figure 7:**
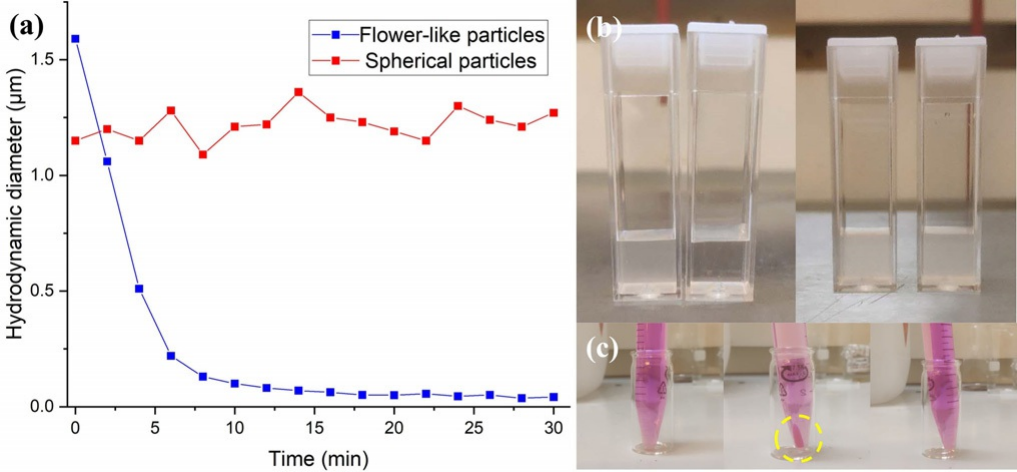
a) Time‐resolved hydrodynamic diameter of the degradation process monitored with DLS. b) Images of the particle suspension before and after degradation of flower‐like particles (left, polymerized in phenyl acetate at 65 °C) and spherical particles (right, polymerized in phenyl acetate at 105 °C). c) Images of (left) the initial Rhodamine B solution (5 μg mL^−1^), (middle) the dispersion after centrifugation (the sedimented particles are circled in yellow), and (right) the dispersion after degradation and centrifugation.

As the disulfide bonds in the cross‐linker are sensitive to a reductive environment, particles prepared in phenyl acetate at 65 °C and 105 °C were subjected to degradation experiments. LC particles were first dispersed in 10 mM KOH solution to prepare a 0.1 mg mL^−1^ particle suspension, and then 2‐mercaptoethanol was added so that its concentration was 1 mM. A rapid decrease in hydrodynamic diameter from 1.59 μm to 0.1 μm indicated the rapid degradation of the flower‐like particles into soluble linear polymers, whereas no apparent change in hydrodynamic diameter was observed for the spherical particles (Figure 7). Such a discrepancy can be attributed to the difference in surface‐to‐volume ratio, even though these particle samples have the same chemical composition and comparable size.

Rhodamine B was used as a tracer dye to demonstrate that the flower‐like particles can be used as redox‐responsive delivery system. The empty particles were first introduced to an aqueous solution of Rhodamine B for an hour (Figure 7 c). After centrifugation of the suspension, the dye was mainly localized in the particle fraction. After redispersion of the loaded particles, 2‐mercaptoethanol was added. After 15 min the solution was centrifuged again: there was no evidence of remaining particles, suggesting complete degradation of the particles (vide supra), indicating that the flower‐like particles can be used as redox‐responsive carrier systems (see Figure S10).

## Conclusion

In this paper, we report the facile synthesis of monodisperse flower‐like particles via one‐step precipitation polymerization of liquid crystalline monomers. By adding ≤10 wt % of cross‐linker **1** to the monomer mixture, a flower‐like morphology was induced. Particle sizes and the number of “petals” in the “flower” were found to mainly depend on the solvent used as reaction medium, whereas the morphology of the particles can be tuned from flower‐like to disk‐shape and further to spherical by elevating the polymerization temperature. A gradual decrease in the molecular order in particles prepared at higher polymerization temperature may contribute to the formation of rounded particles. It was found that the morphology of the particles was formed at the early stage of polymerization, while continued polymerization resulted in the growth of particle size without significantly changing the morphology. Finally, the flower‐like particles were degraded much more rapidly than their spherical counterparts in reductive environments, attributed to their significantly higher surface‐to‐volume ratio.

Our work introduces a facile and scalable preparation of non‐spherical organic particles of a narrow size distribution and may lead to potential applications ranging from superhydrophobic coatings to redox‐responsive carrier systems.

## Conflict of interest

The authors declare no conflict of interest.

## Supporting information

As a service to our authors and readers, this journal provides supporting information supplied by the authors. Such materials are peer reviewed and may be re‐organized for online delivery, but are not copy‐edited or typeset. Technical support issues arising from supporting information (other than missing files) should be addressed to the authors.

Supporting Information
