# Supplementary material for: Flower‐Like Colloidal Particles through Precipitation Polymerization of Redox‐Responsive Liquid Crystals
Source: Angew Chem Int Ed Engl. 2021 Nov 17;60(52):27026–30. doi: 10.1002/anie.202111521 (PMC9298913; doi:10.1002/anie.202111521)
Supplement: Supplementary file 1 — Supporting Information [file ANIE-60-27026-s001.pdf]

Angewandte Chemie
Eine Zeitschrift der Gesellschaft Deutscher Chemiker

Supporting Information

Flower-Like Colloidal Particles through Precipitation Polymerization of Redox-Responsive Liquid Crystals

Xiaohong Liu, Mohammad-Amin Moradi, Tom Bus, Michael G. Debije, Stefan A. F. Bon, Johan P. A. Heuts, and Albert P. H. J. Schenning**

anie_202111521_sm_miscellaneous_information.pdf

Table of Contents

Experimental Procedures 2
Additional Figures 4

Experimental Procedures

Materials

4-(3-acryloxy-propyl-1-oxy)benzoic acid, and 4-(6-acryloxy-hexyl-1-oxy)benzoic acid (**2**) were purchased from Synthon Chemicals, Germany, and recrystallized in ethanol and water before use. Rhodamine B (>95%), 2-Mercaptoethanol (99%), 4-dimethylaminopyridine (DMAP, 99%), pyridine (99%), phenyl acetate (99%), 2,2'-azobis(2-methylpropionitrile) (AIBN,98%), Luperox A75 (benzyl peroxide, BPO, 75%, remainder water), 1,1'-Azobis(cyclohexanecarbonitrile) (ACCN, 98%), Luperox P80 (1,1-bis(tert-butylperoxy)cyclohexane, 80 wt % in benzyl butyl phthalate, technical grade), and tert-butyl peroxybenzoate (98%) were purchased from Sigma-Aldrich. 4-hydroxybenzenethiol was purchased from TCI Europe. 1-Ethyl-3-(3-dimethylaminopropyl)carbodiimide hydrochloride (EDC) was purchased from Fluorochem. All solvents were purchase from Biosolve.

Characterization

Nuclear magnetic resonance (NMR) spectra were recorded on a 400 MHz Bruker Avance III HD spectrometer with tetramethyl silane used as an internal standard. Differential scanning calorimetry (DSC) curves were measured with a DSC Q2000 from TA Instruments. Scanning electron microscopy (SEM) images were taken with a JEOL TM 220 A. The average diameter, coefficient of variation (CV), and circularity were calculated using ImageJ (CV = standard deviation/average diameter; circularity = $4\pi \times$ area / perimeter^2). Transmission electron microscopy (TEM) images were taken with a Tecnai 20 (type Sphera) by FEI operating with a LaB 6 filament at 200 kV. Tomographic tilt series are manually collected from minimum -65° to maximum +65° at 5° steps with Inspect3D software (Thermo Fisher Scientific). Alignment and reconstruction of the series were performed using IMOD. The dry LC particle powders were filled in a 1 mm diameter glass capillary for X-ray diffraction (XRD), which was performed on a Ganesha lab instrument with a GeniX-Cu ultralow divergence source producing X-ray photons with a wavelength of 1.54 Å and a flux of 1×10^8 ph/s. Scattering patterns were collected with a Pilatus 300 K silicon pixel detector with 478 × 619 pixels, each 172 μm^2 in size. Dynamic light scattering (DLS) was measured with Anton Paar Litesizer 500 in backscattering mode at 25 °C. Matric-assisted laser desorption/ionization time-of-flight (MALDI-TOF) measurements were carried out on a Perspective Biosystem Voyager-DE PRO spectrometer by using α-Cyano-4-hydroxycinnamic acid as matrix material.

Experimental procedures

Synthesis of disulfanediylbis(4,1-phenylene) bis(4-(3-(acryloyloxy)propoxy)benzoate) (1)

4-[(4-Hydroxyphenyl)disulfanyl]phenol was synthesized via a published procedure.[1] Briefly, 2 g of 4-hydroxybenzenethiol was added to 8 mL DMSO and the solution was heated to 60 °C for 2 hours. After the reaction, ice water was added to the solution and the mixture was filtered. The pale yellow solid was collected and dried in a vacuum oven overnight (3.91 g, 15.6 mmol, 99%).
4-[(4-Hydroxyphenyl)disulfanyl]phenol (2 g, 8 mmol), 4-(3-acryloxy-propyl-1-oxy)benzoic acid (4.4 g, 17.6 mmol), DMAP (0.391 g, 3.2 mmol), and 30 mL dry dichloromethane (DCM) were added to a round-bottom flask equipped with a magnetic stir bar. The mixture was cooled to 0 °C and EDC (3.8 g, 20 mmol) dissolved in 10 mL DCM was added dropwise. The mixture was stirred at room temperature overnight. After the reaction, the solution was washed with 1 M HCl, saturated $NaHCO_3$, and deionized water. DCM was removed under reduced pressure and the solid recrystallized in ethanol twice to yield the off-white powder product. (2.92 g, 4.08 mmol, 51%)
^{1}H NMR (399 MHz, $CDCl_3$): δ 8.13 (d, *J* = 8.5 Hz, 4H), 7.55 (d, *J* = 8.4 Hz, 4H), 7.17 (d, *J* = 8.4 Hz, 4H), 6.97 (d, *J* = 8.6 Hz, 4H), 6.47 – 6.37 (m, 2H), 6.14 (dd, *J* = 17.3, 10.4 Hz, 2H), 5.89 – 5.81 (m, 2H), 4.38 (t, *J* = 6.3 Hz, 4H), 4.16 (t, *J* = 6.1 Hz, 4H), 2.21 (p, *J* = 6.2 Hz, 4H).
^{13}C NMR (101 MHz, $CDCl_3$): δ 166.12, 164.62, 163.19, 150.64, 134.00, 132.36, 131.02, 129.55, 128.27, 122.61, 121.67, 114.34, 64.70, 61.16, 28.53.
MS (MALDI-TOF): $[M + H]^+$ calculated for $C_{38}H_{35}O_{10}S_2$: 715.17; found $[M + Na]^+$ 737.18, $[M + DMAP]^+$ 837.30. (Figure S3)

It should be noted that monomer **1** can also be synthesized with pyridine instead of DMAP as the catalyst using the same procedure as described above. The H and C NMR spectra of **1** are the same but in the MS spectrum the DMAP adduct no longer present: MS (MALDI-TOF): $[M + H]^+$ calculated for $C_{38}H_{35}O_{10}S_2$: 715.17; found $[M + Na]^+$ 737.17. (Figure S4)

Synthesis of the LC particles

90 mg of **1** and 10 mg of **2** were added to a 50 mL round-bottom flask; the flask was pumped and backfilled with N_2 3 times to remove O_2. The initiator (AIBN (65 °C), BPO (75 °C), ACCN (85 °C and 90 °C), Luperox P80 (95 °C), or tert-butyl peroxybenzoate (105 °C), 4 wt% vs. monomers)) was dissolved in 5 mL solvent and added to the flask. The flask was put into an oil bath preheated to the polymerization temperature for 24 hours. After polymerization, ethanol was added to the suspension, which was then centrifuged at 6500 rpm for 5 minutes. The solvent was removed, and the particles were washed with ethanol 3 times and centrifuged to yield the particles.

Degradation of the LC particles

0.1 mg LC particles were sonicated and dispersed in 1 mL 10 mM KOH solution. A DLS measurement was performed at 25 °C (t = 0 min). Then, 10 µL 0.1 M 2-mercaptoethanol aqueous solution was added to the suspension. A series of DLS measurements were performed at 25 °C with a time interval of 2 mins.

Monomer conversion measurement

The polymerizations were stopped at specific times (2 hours, 4 hours, or overnight (approximately 20 hours)). The particles were separated from the solution by centrifugation (6500 rpm for 5 minutes) and the solution retained. The solutions were carefully concentrated below 40 °C to avoid further polymerization, and ^{1}H- NMR spectra were taken (Figure S8). The integrals of the acrylate peaks (I_1, the sum of three groups at 5.7 to 6.5 ppm, labelled as “a” in Figure S8,) and reference peaks (I_2, at 4.1 to 4.2 ppm, labelled as “b+b’” in Figure S8) were measured, and the monomer conversions in the solutions were calculated by con. = $1 – 2/3 \times I_1/I_2$ (Figure S9 and Table S1).

Absorption and release of Rhodamine B

50 µL 1 mg/mL Rhodamine B aqueous solution, 1 mL 1 mg/mL LC particles (polymerized at 65 °C in phenyl acetate) dispersed in 10 mM KOH aqueous solution were added to 8.95 mL 10 mM KOH aqueous solution. The suspension was left for an hour prior to centrifugation, and the UV-Vis spectrum of the supernatant was measured (red line in Figure S10). The supernatant was returned to the bulk solution, and the particles were redispersed by sonication. 10 µL 1 M 2-mercaptoethanol aqueous solution was added to the suspension, which was centrifuged again after 15 minutes and the UV-Vis spectrum of the supernatant was measured (blue line in Figure S10)

Additional Figures

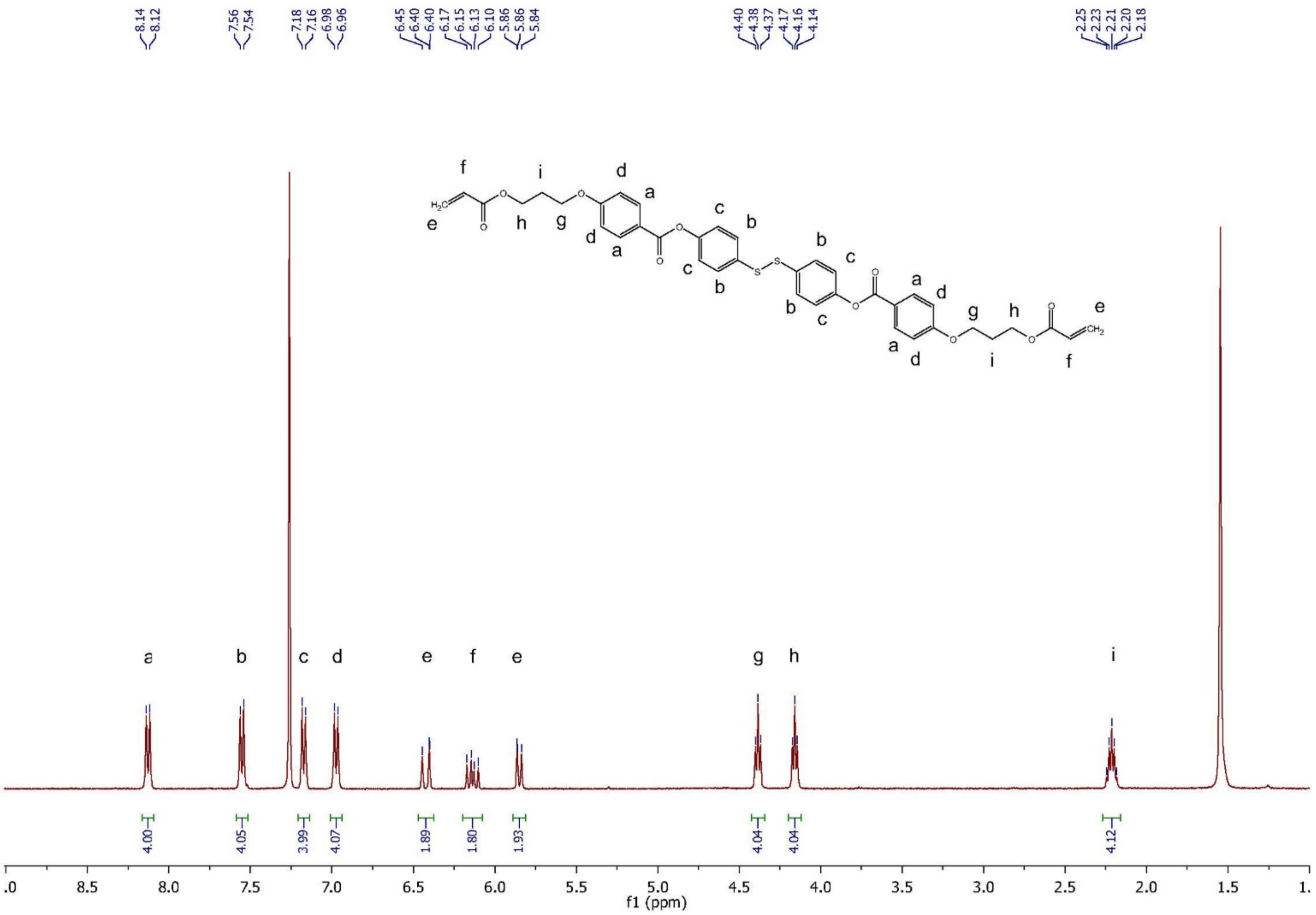

Figure S1. ^{1}H NMR spectrum of monomer **1**.

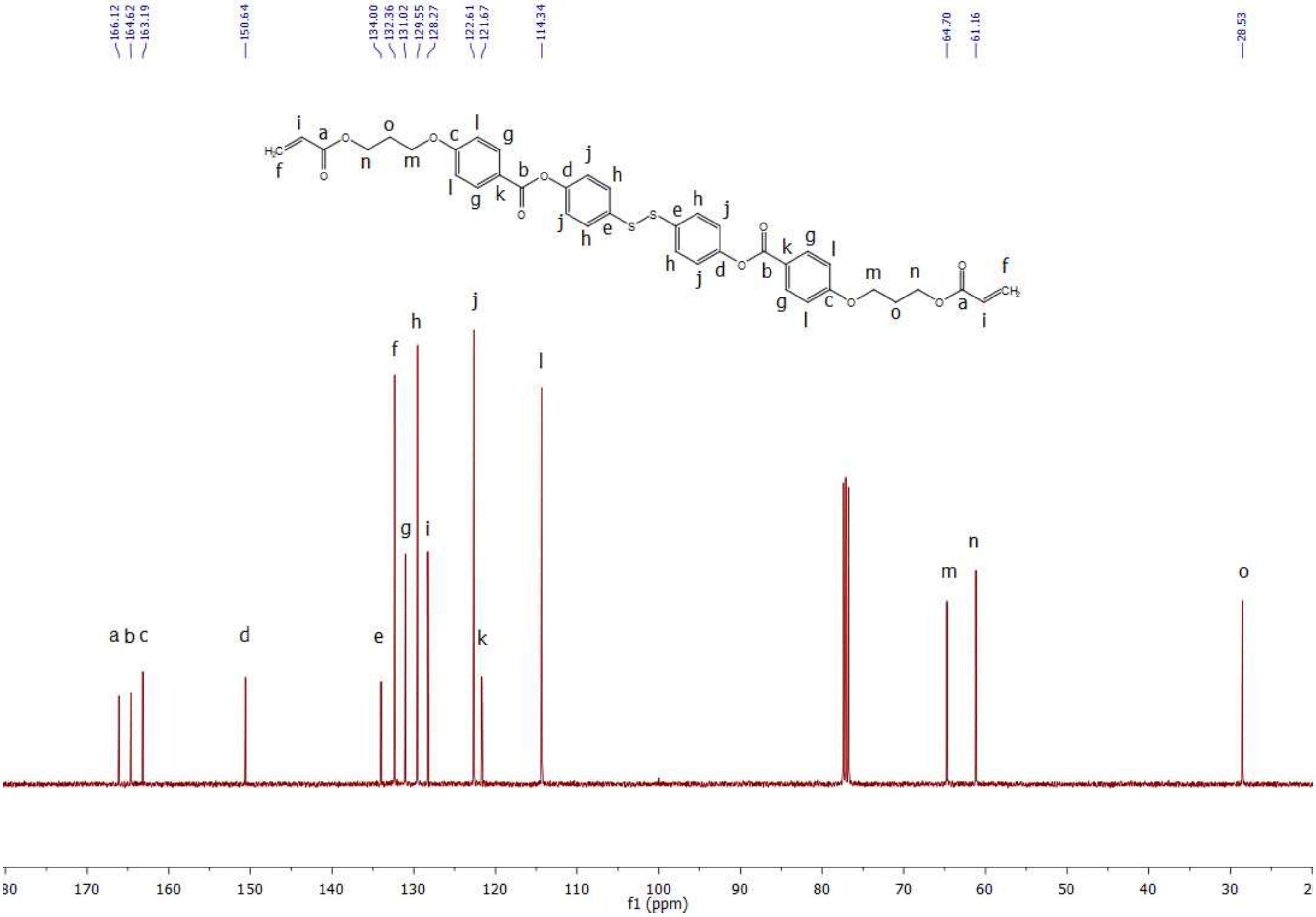

Figure S2. ^{13}C NMR spectrum of monomer **1**.

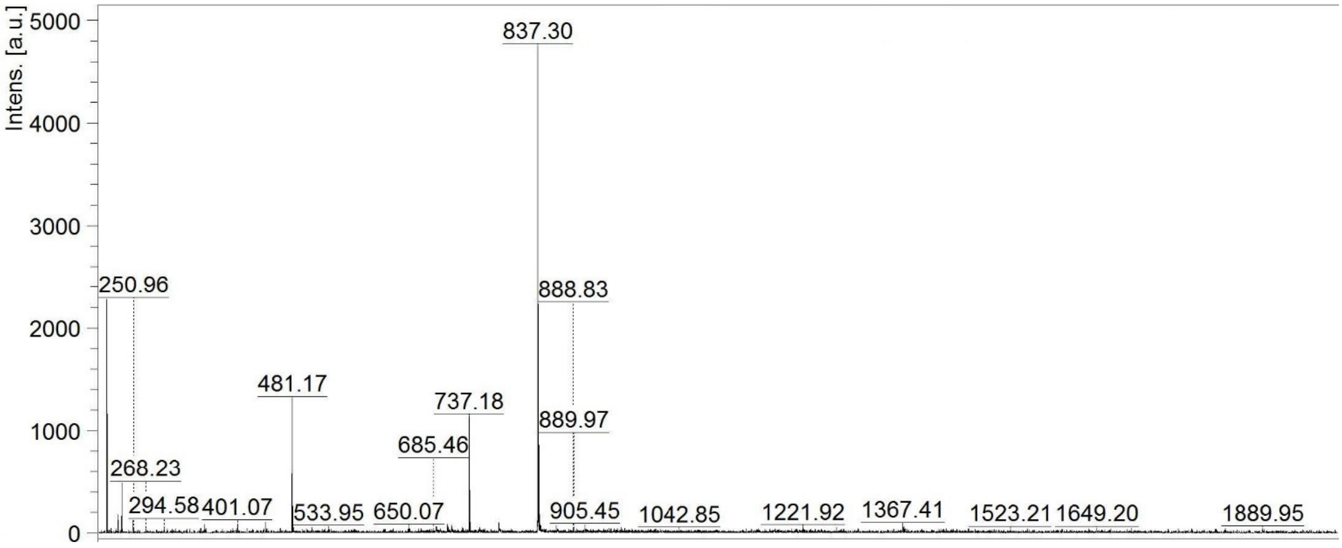

Figure S3. MALDI-TOF spectrum of monomer **1** prepared with DMAP as the catalyst.

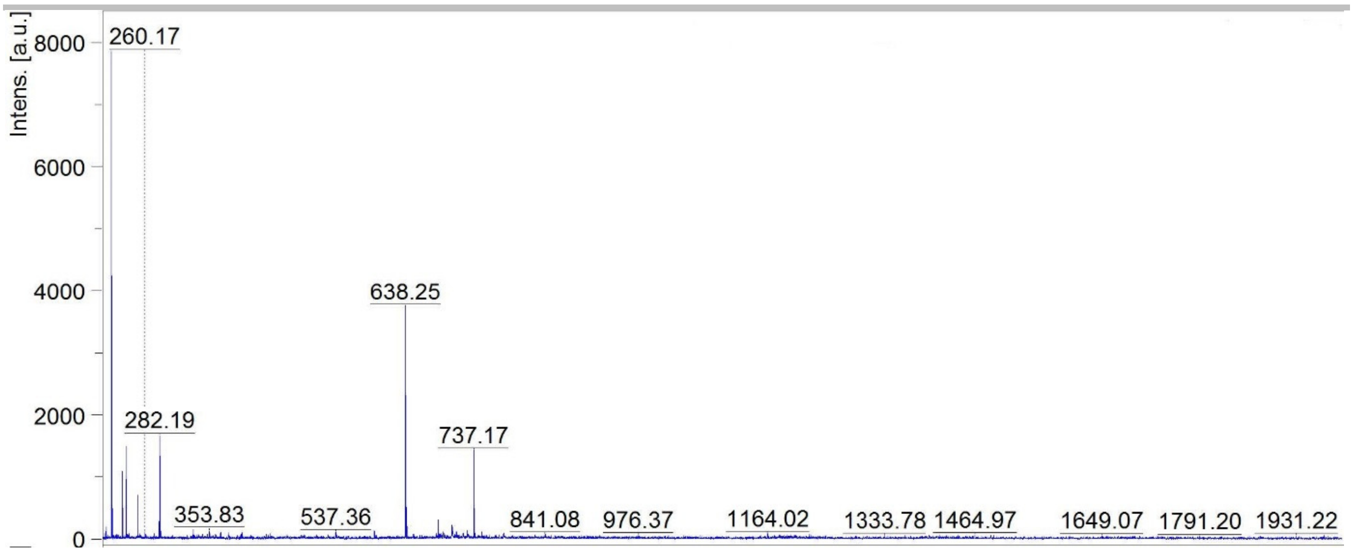

Figure S4. MALDI-TOF spectrum of monomer **1** prepared with pyridine as the catalyst.

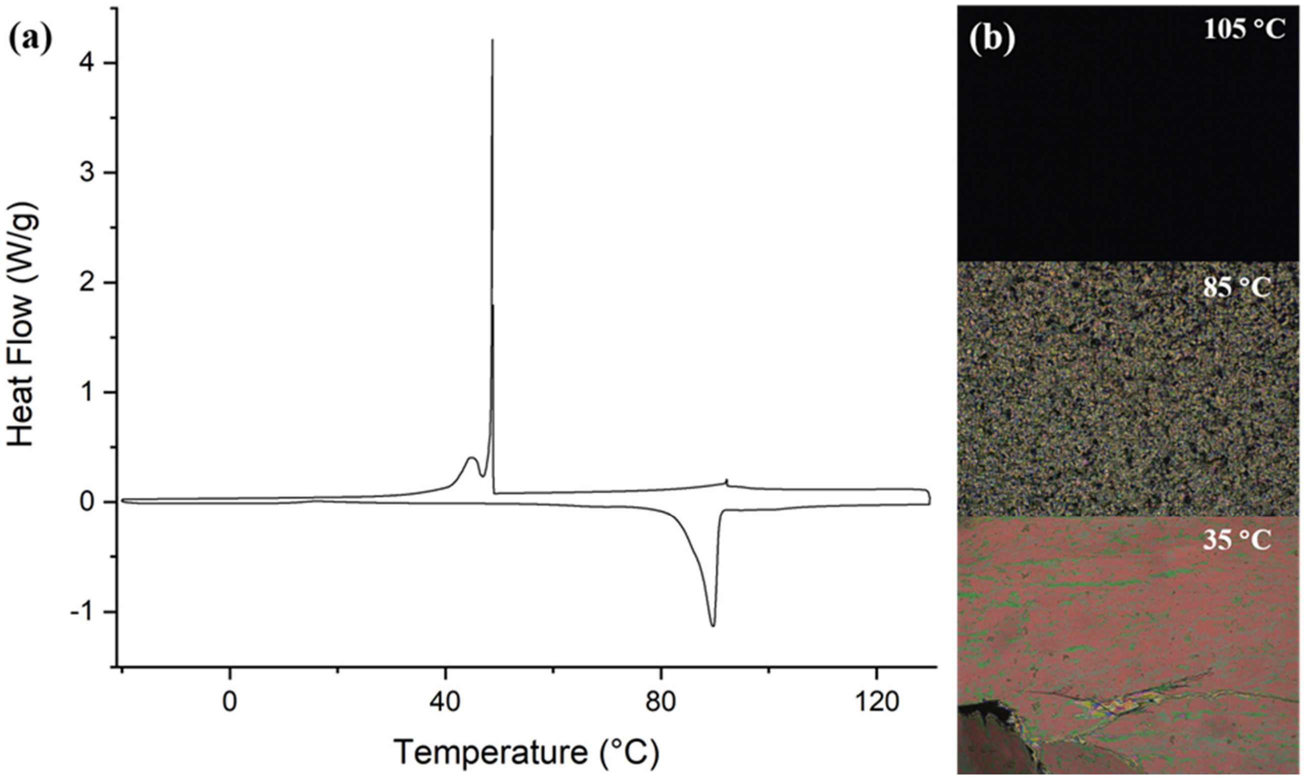

Figure S5. (a) DSC curve of the LC monomer mixture (weight ratio of **1** and **2** = 1:9). The isotropic to LC transition temperature was found around 92 °C, and the LC to crystalline temperature was around 50 °C (exothermal up). (b) POM images of the monomer mixture at 105 °C (isotropic), 85 °C (LC), and 35 °C (crystalline) (planar alignment).

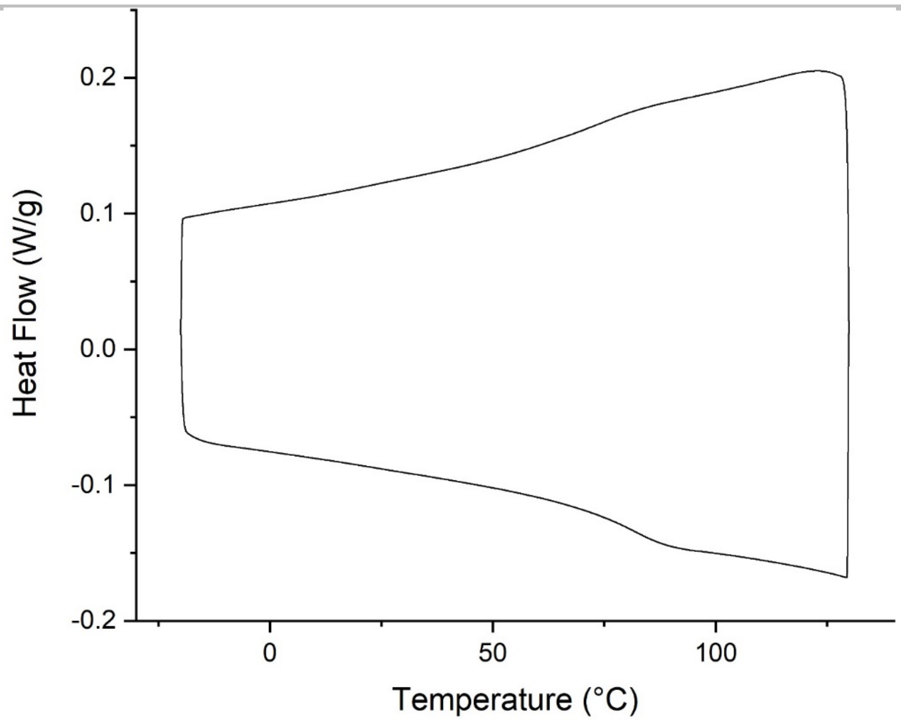

Figure S6. DSC curve of the polymer particles (weight ratio of **1** and **2** = 1:9). The glass transition temperature was found around 82 °C.

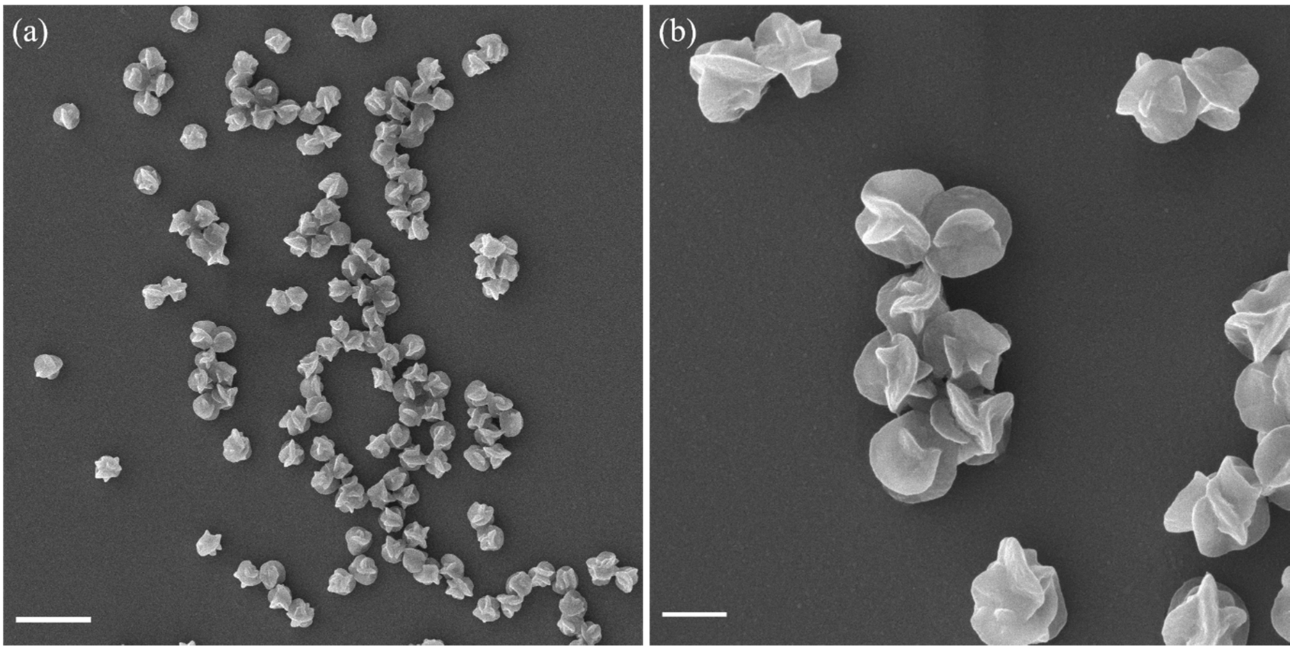

Figure S7. SEM images of LC particles polymerized in phenyl acetate at 65 °C and heated to 105 °C overnight: scale bar = 2 µm (in (a)) and 500 nm (in (b)).

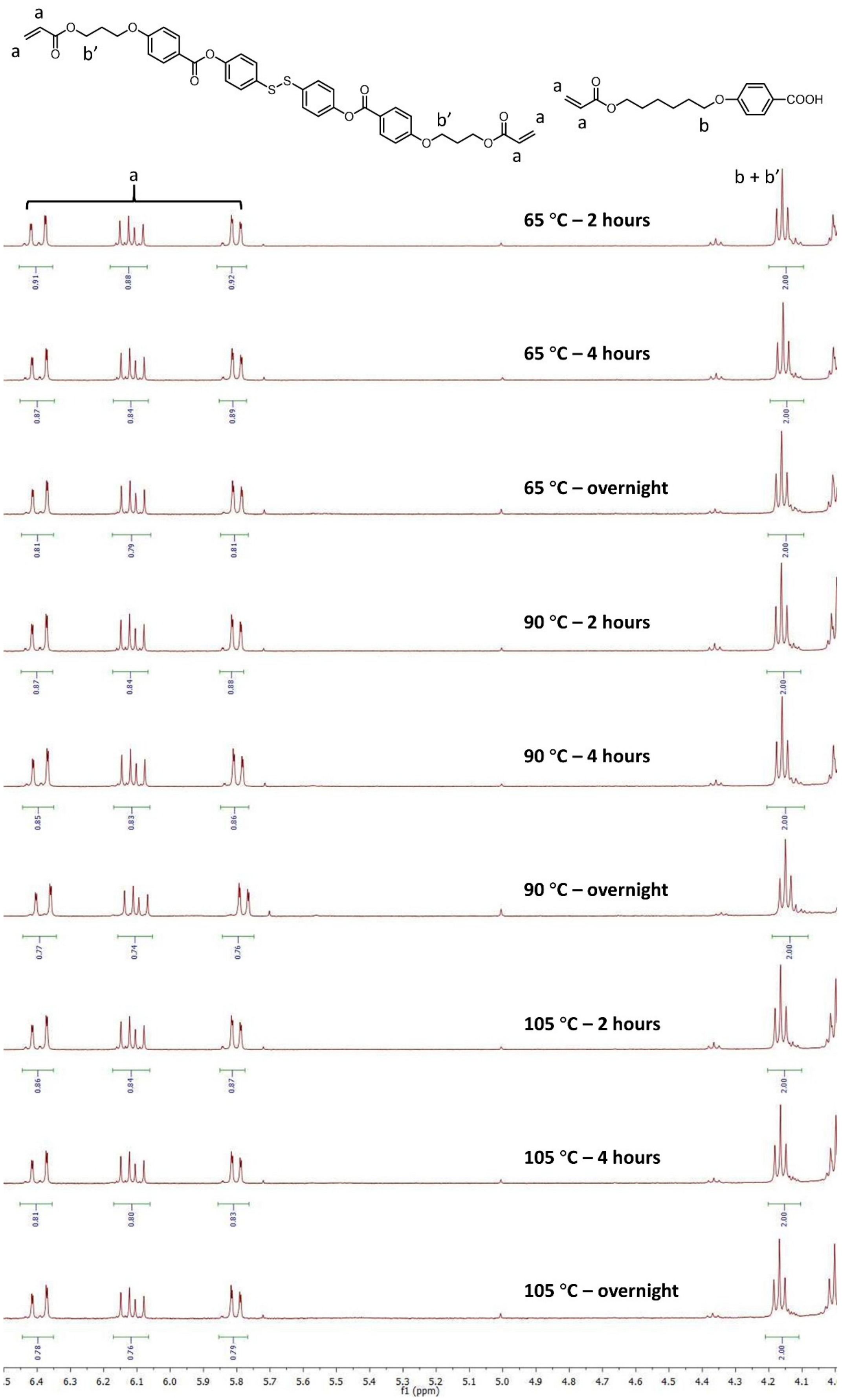

Figure S8. ^{1}H-NMR spectra of the solution polymerized at different temperatures and time intervals. The acrylate peaks were labelled as "a", and the reference peaks were labelled as "b+b'".

Polymerization temperature (°C)	Time	$m_{Particles}$ (mg)	$m_{Soluble\ polymer}$ (mg)	$m_{Unreacted\ monomers}$ (mg)
65	2 hours	8	9	83
	4 hours	20	10	70
	Overnight	52	10	38
90	2 hours	8	13	79
	4 hours	21	12	67
	Overnight	56	11	33
105	2 hours	7	13	80
	4 hours	21	15	64
	Overnight	41	13	46

Table S1. The respective weight of particles, soluble polymers, and unreacted monomers calculated based on Figure S8.

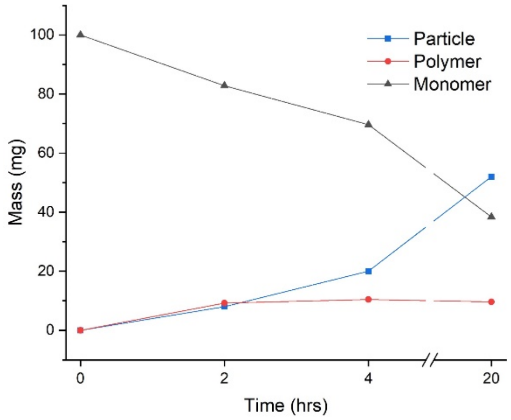

Figure S9. The weight of particles, soluble polymers, and unreacted monomers of polymerization performed at 65 °C calculated based on Figure S8.

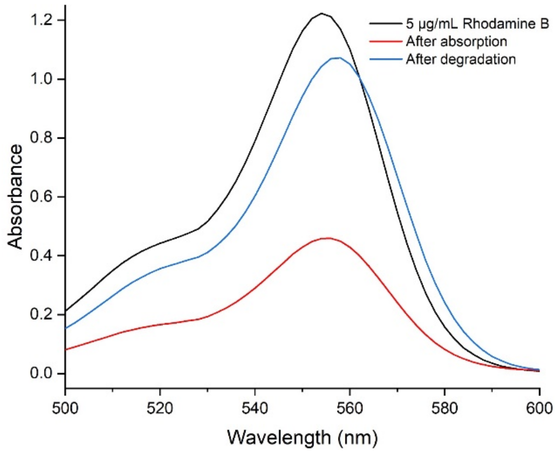

Figure S10. UV-Vis spectra of Rhodamine B solution (black line), the supernatant after adding the flower-like particles (red line), and the supernatant after degradation of the particles upon addition of 2-mercaptoethanol (blue line).
